# References for Ultrasound Staging of Breast Maturation, Tanner Breast Staging, Pubic Hair, and Menarche in Norwegian Girls

**DOI:** 10.1210/clinem/dgaa107

**Published:** 2020-03-06

**Authors:** Ingvild Særvold Bruserud, Mathieu Roelants, Ninnie Helén Bakken Oehme, Andre Madsen, Geir Egil Eide, Robert Bjerknes, Karen Rosendahl, Petur B Juliusson

**Affiliations:** 1 Department of Clinical Science, University of Bergen, Bergen, Norway; 2 Department of Paediatrics, Haukeland University Hospital, Bergen, Norway; 3 Environment and Health, Department of Public Health and Primary Care, KU Leuven–University of Leuven, Leuven, Belgium; 4 Hormone Laboratory, Haukeland University Hospital, Bergen, Norway; 5 Centre for Clinical Research, Haukeland University Hospital, Bergen, Norway; 6 Department of Global Public Health and Primary Care, University of Bergen, Bergen, Norway; 7 Department of Radiology, University Hospital of North Norway, Tromsø, Norway; 8 Department of Clinical Medicine, University in Tromsø, The Artic University of Norway, Norway; 9 Department of Health Registries, Norwegian institute of Public Health, Bergen, Norway

**Keywords:** puberty, breast development, menarche, female

## Abstract

**Context:**

Discriminating adipose and glandular tissue is challenging when clinically assessing breast development. Ultrasound facilitates staging of pubertal breast maturation (US B), but has not been systematically compared to Tanner breast (Tanner B) staging, and no normative data have been reported.

**Objective:**

To present normative references for US B along with references for Tanner B, pubic hair (PH), and menarche.

**Design, Setting, and Participants:**

A cross-sectional sample of 703 healthy girls aged 6 to 16 years were examined.

**Main Outcome Measures:**

Breast development was determined with US B and Tanner B staging. Tanner PH and menarcheal status were recorded. The age distributions of entry in US B, Tanner B, and PH stages and menarche were estimated with generalized linear and generalized additive models with a probit link. Method agreement was tested with weighted Cohen’s kappa.

**Results:**

The median (±2SD) ages for thelarche, US B2 and Tanner B2, were 10.2 (7.7, 12.8) and 10.4 (8.0, 12.7) years. The median (±2SD) ages at Tanner PH2 and menarche were 10.9 (8.5, 13.3) and 12.7 (11.0, 16.2) years. Cohen’s kappa of agreement (95% confidence interval) between US B and Tanner B was 0.87 (0.85–0.88). When the methods disagreed, US B was usually more advanced.

**Conclusion:**

Thelarche occurred at a slightly younger age when assessed with ultrasound compared to clinical Tanner staging, although the 2 methods had a very good agreement when determining pubertal breast maturation. A significant decrease of 2.8 months in age at menarche was observed during the past decade in Norwegian girls.

Breast development in girls is usually defined clinically by the 5-stage scale as described by Marshall and Tanner in 1969 (1,2). The Tanner breast (Tanner B) scale ranges from B1 (prepubertal) to B5 (mature), in which stage B2 marks the first appearance of glandular breast tissue (thelarche) and thus the start of central puberty. Clinical breast staging relies on visual inspection and palpation to distinguish the glandular breast tissue from adipose tissue, but this approach is often perceived as challenging. With ultrasound (US), we, and others, have been able to identify distinctive breast developmental stages based on changes in the appearance and relative amount of glandular, adipose and fibrous tissues (3–5). Despite being a promising method, US staging of the breast has not yet been systematically compared with clinical Tanner B staging in a large developmentally diverse sample, and no normative references have yet been published.

In Norway and other northern European countries, only marginal changes in age at menarche have been reported during the last decades (6–10). However, data from Denmark and the United States have shown that the onset of breast development might be advancing toward younger ages (7,11,12). A worldwide secular trend toward younger ages at thelarche according to race/ethnicity and geography was also shown in a recent systematic review (13). Accordingly, these studies suggest an asymmetrical secular trend of earlier breast development, while age at menarche remains more stable. Early puberty, and early menarche in particular, have been associated with increased all-cause mortality (14), a higher risk of breast cancer (15), cardiovascular disease (16), and mental health problems (17). It has been suggested that this secular trend may be caused by overweight or endocrine disrupting chemicals (18,19).

The present study is a part of the Bergen Growth Study 2 (BGS 2), which is the first large-scale pubertal reference study conducted in Norway. In addition to data on menarche and traditional Tanner B and pubic hair (PH) stages, we collected data on breast developmental stage assessed with US (US B) as a novel methodological approach. The aim of the current work was to provide descriptive reference ranges for these phenotypic traits in a large sample of contemporary girls living in Norway, to compare both methods of staging breast development, and to assess recent changes in age at menarche.

## Materials and Methods

### Study design and participants

A total of 1349 girls between 6 and 16 years of age from 6 randomly selected public schools in Bergen municipality, Norway, were invited to participate. Bergen is the second largest city in Norway and the demographic profile and living conditions are very similar to those in the country at large (20). Parental consent was received prior to examination from 678 girls (50.3%) from January to June 2016. The participation rates varied with age, from 52.1% before 12 years to 43.4% above 13 years, and between schools, from 29.1% to 59.4%. In addition, data on breast development from 57 girls examined in February 2017 for a reliability study were included in the analysis. Details from this study have been published previously (5). A parental questionnaire with information on chronic illness, parental origin, socioeconomic status, and menarche was obtained from 482 (68.6%) girls. Five girls were absent on the day of examination, and 27 girls were excluded due to a disease likely to affect growth or maturation, leaving 703 girls eligible for the analysis. Of 466 girls with known origin of both parents, 374 (80.2%) were Norwegian, 408 (88.9%) European, and 51 girls (11.1%) had 1 or 2 non-European parents, mostly from Asia (*n* = 30), Africa (*n* = 12), or South America (*n* = 7) (Supplementary Table 1, all supplementary materials and figures are located in a digital data repository (21)). The analyses are based on all girls, regardless of their origin. The highest educational level of both parents was used as an indicator of socioeconomic status. This level was classified as primary education (9 years of school), secondary education (12 years of school), or higher education (college degree). Demographics of the study populations from Bergen Growth Study 1 (BGS 1) and BGS 2 are summarized in Supplementary Table 1 (21).

The median (range) age in the final sample was 10.7 (6.1–16.2) years. Due to missing data (menarche was not recorded in 3 girls, 3 girls declined examination of the breast, and 81 declined examination of PH, and the US image could not be retrieved for off-site scoring in 4 girls), the pubertal references are based on 696 observations of US B, 700 of Tanner B, and 643 of menarche. Data on the Tanner PH stage were available for 372 girls as further explained in the following discussion.

### Examinations

The girls were examined during school hours. All examinations were performed by a single female nurse (ISB), who was trained in US staging of breast development by an experienced pediatric radiologist (KR) before and at the start of the actual data collection. Pubertal development was assessed with the girl in the supine position. The US examinations were performed using a Sonosite Edge machine (Fujifilm SonoSite, US) with a 15–6 MHz (5 cm) linear probe. Based on a standard mid-sagittal section, breast development was scored on a scale from 0 to 5, consistent with our previously published descriptions (5). US breast staging was conducted based on radiologic characteristics (Supplementary Figure 1, (21)). In brief, a small, hypoechoic retro-areolar mass is scored as US B0, while a hyperechoic area (with or without a small hypoechoic mass) is US B1. Both US B0 and US B1 are considered prepubertal. US B2 is characterized by the presence of hyperechoic retro-areolar glandular breast tissue with a round or star-shaped hypoechoic center. This stage is assumed to mark the start of puberty (3,5). US B3 is characterized by a larger, spider-shaped hypoechoic center, becoming increasingly roundish in US B4 (5). The mature US B5 is characterized by heterogeneous glandular breast tissue without the hypoechoic center observed in US B2 to B4. The left breast was examined according to the protocol. Due to logistic reasons, the US stage was determined at a later time in 166 girls, by the same examiner, based on the standardized US images. This was done to avoid the girls becoming uncomfortable due to prolonged exposure. Based on a reassessment of 120 saved images, we determined that this had little impact on the results. Intra- and inter-observer agreement of US B staging was determined in an additional study of 57 girls (5). Cohen’s kappa was estimated at 0.84 and 0.71, respectively, which is commonly considered good to very good agreement (22).

The development of the breasts and PH was clinically scored on a scale from 1 to 5 (B1–B5 and PH1–PH5) according to the method described by Tanner (1), which is based on a visual inspection, although palpation of the breast was included in this procedure. Pubertal onset is defined as Tanner B2, which is characterized by the visual and/or palpable appearance of glandular tissue. The left breast was always examined, however, in 3 cases the right breast was found more advanced according to Tanner B staging and therefore used in the references (Tanner B staging recorded additionally on the right side in 453 girls). A subsample of 453 girls were asked if the examiner could assess the Tanner PH by visual inspection, to which 372 (82.1%) assented. Because of assumed time constrains, Tanner B staging of the right breast and Tanner PH staging were not included in the protocol at the start of the study but were only added and systematically recorded from February 22 until the end of the data collection. Tanner PH2 marks the first appearance of PH (1,2). All participants were asked if they had experienced their first menstruation (menarche) and the month/year, if applicable. Missing data on menarche were completed with data from the parental questionnaire for 16 girls. Two girls above 15 years of age were encouraged to see their general practitioner due to primary amenorrhea.

Height was measured in the standing position with a Harpenden Portable Stadiometer (Holtain Ltd Crosswell, UK) and recorded to the nearest 0.1 cm. Weight was measured in light clothing with an electronic scale (Tanita MC-780MA, Tanita Corp. of America, Inc. Illinois, US) with a precision of 0.1 kg. Body mass index (BMI) was calculated by dividing weight (in kg) by the square of height (in meters). The BMI was available for 645 (91.7%) girls of whom 14.6% were overweight (including obesity) and 7.3% were underweight according to the definitions from the International Obesity Task Force (23,24).

### Statistical analysis

Age at menarche or entry into a certain puberty stage was estimated from age and pubertal status of the girls with a generalized linear model (probit regression) and with a nonparametric generalized additive model (GAM) for binary outcomes with probit link. The methods differ by the assumption of a normal age distribution (probit) or not (GAM). The probit and GAM curves were estimated for each consecutive stage separately. The degree of smoothing of the GAM was determined by generalized cross-validation and models were checked with diagnostic plots using the mgcv package (25) in R version 3.5 (R Foundation for Statistical Computing, Vienna, Austria, 2018). Primarily, the median, percentiles, and equivalent z-scores of age at entry from the nonparametric additive model are reported in the tables. In addition, we report the mean and standard deviation (SD) from the parametric probit models to allow a comparison with data from other studies. In addition to reference data based on all eligible girls, we estimated age at menarche and transition to US B2, Tanner B2, and Tanner PH2 in the subgroup of girls with a documented Norwegian or non-Norwegian origin. The statistical significance of differences between Norwegian and non-Norwegian girls was tested with logistic regression using age as a covariate and origin (Norwegian, non-Norwegian) as the variable of interest. This analysis was limited to girls with a documented origin only.

Agreement between US B and Tanner B staging was analyzed with simple Cohen’s kappa and Cohen’s kappa test with linear weights. The kappa statistic gives a coefficient between 0 (no agreement) and 1 (perfect agreement) (22). For these comparisons, the US B0 and B1 stages were collapsed into a single prepubertal stage. Separate analysis of agreement and percentage concordance between the methods were done for girls with normal weight and overweight (including obesity).

To investigate if age at menarche had advanced in the Bergen municipality since the first Bergen Growth Study (BGS 1), conducted in 2003 to 2006, we compared girls with a documented Norwegian or Nordic (1.5%) background in both studies to avoid interference from demographic changes in the population. Details from BGS 1 have been published elsewhere (10). In brief, girls from 8 to 15.5 years (*n* = 1481) were asked if they had experienced their menarche, and this was the only pubertal marker recorded in BGS 1. The occurrence of menarche was compared between both studies with logistic regression using age, BMI *z*-score, and parental educational level as covariates. Logistic regression, and agreement analyses were done in Statistical Package for Social Sciences (SPSS version 24) and R (version 3.5).

### Ethical considerations

The project was approved by the Regional Committee for Medical and Health Research Ethics West Norway (number 2015/128/REK Vest). Written informed consent was obtained from a parent or legal guardian of each participant and also from the participants 12 years and older. All girls received age-appropriate information in writing and orally by the study nurse ahead of participation. Assent from the participant was an additional requirement for inclusion. All participants received a cinema ticket for their collaboration.

## Results

**Figure 1.**
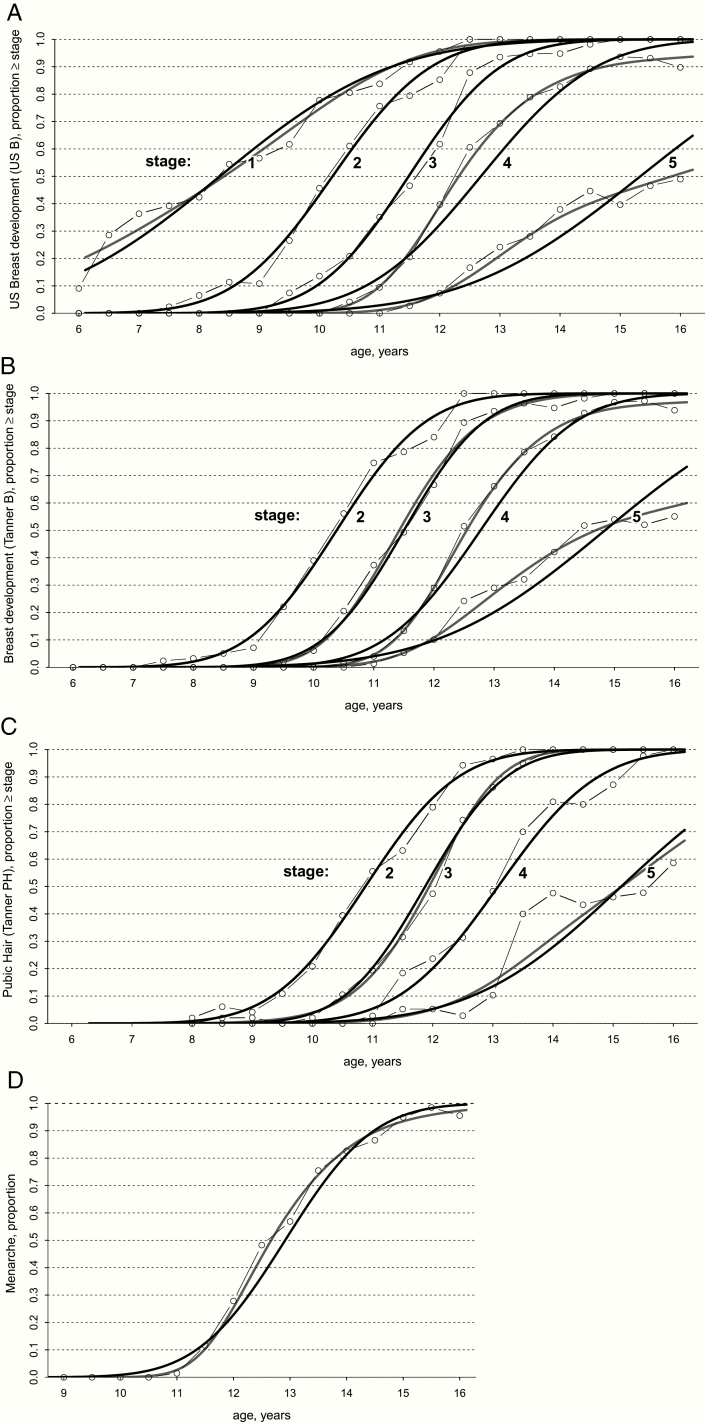
Age reference curves. Age distribution of pubertal development in 6- to 16-year-old girls living in Norway. **A:** Stages of breast development with ultrasound (*n* = 696); **B**: Breast development stage by Tanner stage (*n* = 700); **C:** Pubic hair (PH) according to Tanner (*n* = 372); **D:** Menarche (*n* = 643). Black lines show the parametric probit model, assuming a Gaussian distribution of the age at transition; grey lines show the corresponding curves of the nonparametric generalized additive model with a probit link; connected dots show the cumulative proportion of girls who have reached a particular pubertal stage in overlapping one-year intervals.

The medians and equivalent *z*-scores, means, and SD for age at entry into consecutive US B, Tanner B and PH stages, and menarche are presented in Table 1. Traditional age percentiles of all pubertal markers are provided in Supplemental Table 2 (21). Reference quantiles were calculated nonparametrically from the cumulative distribution estimated with a GAM, which in some cases coincided with the normal distribution as shown in Figs. 1A–D.

**Table 1.** Age References for Breast Development, Pubic Hair and Menarche: Median and Distribution (–2, –1, 1, 2 SD) of the Age (in Years) at Attainment of the Specified Stage

	Generalized Additive Model Age at Attainment					Probit
Pubertal marker	–2 SD	–1 SD	Median	1 SD	2 SD	Mean (SD) age
**Breast (ultrasound)**						
US B1	NA	5.6^a^	8.3	10.7	12.4	8.3 (2.2)
S B2	7.7	8.9	10.2	11.5	12.8	10.2 (1.3)
US B3	9.1	10.3	11.5	12.7	13.9	11.5 (1.2)
US B4	10.4	11.3	12.3	13.9	NA	12.7 (1.5)
US B5	11.3	12.7	15.9	NA	NA	15.3 (2.3)
**Breast (Tanner)**						
B2	8.0	9.2	10.4	11.6	12.7	10.4 (1.2)
B3	9.5	10.4	11.4	12.5	13.8	11.5 (1.1)
B4	10.7	11.6	12.5	13.8	NA	12.8 (1.3)
B5	11.1	12.3	14.7	NA	NA	14.8 (2.2)
**Pubic Hair (Tanner)**						
PH2	8.5	9.7	10.9	12.1	13.3	10.9 (1.2)
PH3	9.7	10.9	11.9	12.9	13.7	11.9 (1.1)
PH4	10.5	11.8	13.1	14.4	15.7	13.1 (1.3)
PH5	11.4	13.0	15.1	NA	NA	15.1 (1.9)
**Menarche**	11.0	11.7	12.7	14.1	16.2	12.9 (1.2)

Distribution of age at entry in consecutive stages of breast development measured with ultrasound (US B; *n* = 696), breast development according to Tanner B (*n* = 700), Pubic hair (PH) according to Tanner (*n* = 372) and menarche (*n* = 643) in 6- to 16-year-old healthy girls in Bergen, Norway. Median age and ±1 or 2 reference limits were estimated with nonparametric generalized additive models. Mean (SD) ages were estimated with probit regression.

*Abbreviations*: B, breast; NA, not applicable (outside the data range); PH, pubic hair; SD, standard deviation; US B, ultrasound-based breast stage.

^a^Extrapolated age that falls just below the data range.

The distribution of age at onset of breast development measured with US was slightly ahead of the Tanner method. However, the opposite was true for the higher maturational stages, where the age at transition with the Tanner method was ahead of the assessment with US. The mean age (SD) of thelarche was 10.2 (1.3) years with US (US B2), and 10.4 (1.2) years with the Tanner method (Tanner B2). The lower end of the normal range defined by a *z*-score of –2 SD was 7.7 and 8.0 years, respectively, while the corresponding upper limits were 12.8 and 12.7 years.

The breast developmental stage assessed with US and the Tanner method was concordant in 551 of 695 (79.3%) girls for whom both assessments were available (kappa = 0.87; 95% confidence interval [CI]; 0.85–0.89) (Table 2). When dichotomizing the breast developmental stage into thelarche or not thelarche, the agreement was very good (kappa = 0.94; 95% CI; 0.91–0.96). US identified breast development (US B ≥2) in 18 girls in whom the mammary gland was not palpable (corresponding to Tanner B1). Opposite, three girls had clinical signs of pubertal breast development (Tanner ≥B2) with no glandular breast tissue visible on US (US B0 or US B1). The kappa coefficients when comparing the 2 methods were 0.88 (95% CI 0.86–0.91) and 0.85 (95% CI: 0.79–0.90) for girls with normal and overweight/obesity, respectively. Full concordance between US B and Tanner B staging was found in 81.6% and 73.4% of girls with normal weight and overweight/obesity, respectively.

**Table 2.** Distribution of Breast Development with Ultrasound and Tanner Staging

	Breast Development With Ultrasound						
Tanner stage	US B0	US B1	US B2	US B3	US B4	US B5	Total
B1	**166**	**129**	18	0	0	0	313
B2	1	2	**63**	12	2	0	80
B3	0	0	13	**43**	25	2	83
B4	0	0	0	15	**77**	16	108
B5	0	0	0	5	33	**73**	111
**Total**	167	131	94	75	137	91	695

Agreement of the breast developmental stage according to Tanner (B1 to B5) and ultrasound examination (US B0 to B5) in 695 6- to 16-year-old girls in Bergen, Norway. US B0 and US B1 and Tanner B1 are prepubertal stages; stages US B0 and US B1 are merged for the analysis of method agreement.

Abbreviations: US B, ultrasound-based breast stage.

Pubic hair development (Tanner ≥PH2) was observed in 182 of 372 (48.9%) girls. The mean (SD) age at Tanner PH2 was estimated as 10.9 (1.2) years. The age distribution of each Tanner PH stage was close to normal (Fig. 1C).

In total, 193 of 643 (30.0%) girls had experienced menarche. The median (±2 SD) age at menarche was 12.7 (11.0–16.2) years. This is 2.5 years after thelarche according to the US method (US B2) and 2.3 years after thelarche according to the Tanner method (B2). A comparison of the probit model and the GAM indicated a positive skewness in the distribution of age at menarche (Fig. 1D).

The onset of all pubertal markers occurred earlier in girls with a non-Norwegian origin (*n* = 92) compared to girls with two parents of Norwegian origin (*n* = 374), in the subset of girls with known origin (74%) (Supplemental Table 3 (21)). The difference was only statistically significant for the start of breast development according to US (*P* < .05). Compared to girls from BGS 1, mean (SD) age at menarche in girls of Norwegian, including Nordic, origin had significantly declined from 13.3 (1.7) years (not previously published) in 2006 to 13.1 (1.2) years in 2016 (*P* < 0.05). The difference in occurrence of menarche between both studies remained statistically significant (odds ratio 2.0; 95% CI 1.1–3.6; *P* = .016), when accounting for the BMI *z*-score and parental educational level. Parental education was not retained in the final models because it was not significantly associated with menarche in either study. The age distribution in both studies is shown in Supplementary Figure 2 (21) for all girls in the study and in Supplementary Figure 3 (21) for girls of Norwegian/Nordic origin.

## Discussion

In this study, we describe female pubertal development using a large contemporary sample of healthy girls living in Norway. The results are summarized as age percentiles, which can serve as normative reference intervals for pubertal milestones in the Norwegian and other comparable populations. Up-to-date references are important to support clinical decision-making. In addition to the traditional Tanner stages, we have also documented breast development based on US examination of glandular breast tissue. Our results show a high degree of concordance between the 2 methods, but the start of breast development is detected almost 2 months earlier with US staging. Finally, our results indicate that there might be an ongoing decrease in the age at menarche in the Norwegian population.

The principle of assessing breast development with US examination of the mammary gland has been proposed by several authors (4,5,26–28), but US staging of breast development was not yet compared with clinical staging in a large sample of healthy pubescent girls, and no reference data were available. The 4 studies that could be identified were small (<80 subjects) or based on a heterogeneous clinical sample (4,26–28). US staging is a promising technique with a good intra- and inter-observer reliability (5) that may overcome several known limitations of the visual and palpatory method described by Tanner. In the present study, we have shown that both methods agree well with each other in a large sample of girls at various stages of their development, also when stratifying by weight category. The kappa statistic showed good agreement over all five stages and very good agreement when girls were classified as having thelarche or not. The biggest discrepancy was observed between Tanner B5 and US staging, but in most cases, this difference was 1 stage only. This may be due to the statistical model because only about half of the girls have reached stage 5 by the end of the age range of girls included in the study (differences are smaller at –1 or –2 SD) but could also reflect a discrepancy between the size of the breast and maturation of the gland as visualized with US or reflect an overlap of the characteristics of stages 4 and 5 with the Tanner and US methods.

However, the latter comparison also indicated that the US method is probably more sensitive to pick up changes that are consistent with the start of pubertal breast development. In addition, US examination identifies two distinct developmental stages (denoted as US B0 and US B1) in girls who are prepubertal (B1) according to the Tanner method. This has not been acknowledged in previous studies by other authors (5). Both these stages are considered by us as prepubertal and future research will indicate to what extent they correlate with physiological and hormonal changes. For the comparison of the US and Tanner methods both prepubertal stages were merged.

The mean age at thelarche according to the Tanner method (B2) was 10.4 years with an SD of 1.2 years. While no historical data are available for the Norwegian population, other studies reported a substantial decline in onset of breast development (Tanner B2) from 10.9 years in 1993 to 9.9 years in 2008 in Danish girls living in the Copenhagen area (7). However, a more recent population-based longitudinal study of self-reported onset of breast development found a mean age at thelarche of 10.4 years, which is more comparable to our findings (29). A declining age at thelarche has also been reported in the United States (11,30) and in the United Kingdom (9), but studies from Belgium (6) and the Netherlands(31) reported a mean age at thelarche of 10.7 years, without an apparent trend toward younger ages. The lower reference limit (–2 SD) for age at attaining Tanner B2 was 8.0 years in the current study. Overall, these findings suggest that the onset of breast development in Norwegian girls is neither earlier nor later when compared to other northern European countries and that the current age limit of precocious puberty in girls (ie, start of breast development before 8 years) can be maintained, as is the case in other countries (6,32). Likewise, we reported a mean (SD) age of the start of PH development (Tanner PH2) of 10.9 (1.2) years, which corresponds well with the mean ages of 11.0 to 11.1 years reported in northern European populations (6,7,31).

Mean age at menarche fluctuates within a narrow range from 12.9 to 13.1 years in most studies from northern Europe (6,7,9,29,33). Although age at menarche has been remarkably stable for more than half a century, a small but statistically significant decline has been reported in Denmark and the Netherlands (0.3 and 0.1 years, respectively) (7,33). A recent study from the United States reported an overall median age at menarche of 12.3 years and in white girls 12.7 years (12), which is only a modest change from a previous U.S. study where the age at menarche in white girls was 12.9 years (11). In the current study, we found a median age at menarche of 12.7 years, which is in line with the white girls from the United States, but earlier than the cited northern European studies. However, we previously reported an overall median age at menarche of 13.1 years in BGS 1 from 2006 (10). The age at menarche in Norway has been stable just above 13 years during the last 50 years (10,34). To rule out possible interference from recent changes in the composition of the target population such as origin, BMI, and socioeconomic background, we compared a subsample of girls with a documented Norwegian origin from both studies and included BMI and parental educational level in the analysis. Parental education was not significantly associated with age at menarche, but there was a significant effect of the BMI as was documented before (10). The statistically significant decrease with 2.8 months in age at menarche since 2006 was however not affected by the inclusion of the BMI *z*-score in the models because the BMI distribution was highly comparable in both studies. An increased exposure to endocrine disruptive chemicals remains a possible hypothesis, to which we cannot offer a conclusive answer in the current analysis (19). Given the potential importance of this finding, we believe it should be confirmed in a larger population-based study.

For the descriptive reference ranges of menarche and secondary sexual characteristics we have included all girls regardless their origin in the reference sample because their impact on the reference limits is relatively small from a clinical perspective, and implementation of a single reference might be more feasible. Establishing and adapting reference curves to fit an increasingly demographically heterogenic composition where growth and puberty timing of the native population is different to that of immigrant populations remain an epidemiological challenge.

Although the BGS 2 included a large number of children at or near puberty and covers many aspects of pubertal development, some limitations should be noted. The schools were randomly selected and cover various socioeconomic conditions, but they were all situated in mostly urban areas of the municipality of Bergen. The prevalence of overweight (including obesity) of 14.6% in our study is slightly lower than, but still comparable to, data from the first Bergen growth study (17.7%) (35) and to data from a nationally representative sample of 9-year-old girls (17%) (36). Including girls from different Norwegian regions would have increased generalizability but would also have introduced logistic challenges and could have negatively impacted the standardization of measurement procedures. The recruitment strategy is also a potential source of selection bias, that is, when girls with a relatively early or late puberty are more reluctant to participate. Our study sample is representative of the Norwegian population with respect to origin (37). Girls of non-Norwegian origin were included in the analysis since this reflects the natural variation in the target population. Although this increases the applicability of the references in the Norwegian population at large, it may also hamper the comparability of the data with other nations or future studies. For this reason, we also provided references based on a subsample of girls with a Norwegian background as supplementary material (21). Our knowledge about the nonparticipants is limited to school and grade. However, there was a slightly lesser participation rate in schools with a larger number of children with a non-Norwegian background. Increased nonparticipation in these schools may have been caused by language barriers or cultural preferences of parents.

One of the potential advantages of US staging of breast development relates to difficulties with Tanner staging in girls with obesity. We observed that the agreement and concordance between the two breast staging methods were slightly lower among the girls with overweight and obesity and that the clinical assessment, Tanner B, showed a more mature stage compared to the US B in more cases among the girls with overweight than in the normal weight group (data not shown). A higher prevalence of girls with Tanner B2 would have been preferable to further examine the relation between US B and Tanner B around the clinical stage that defines pubertal onset. Another limitation is the low response, even though we consider the participation rate of 50% as relatively high for this particular type of study. Information on health and ethnicity were only known when the questionnaire was answered and returned by parents (69%), which may have resulted in the inclusion of some girls with relevant pathology unknown to us. However, the prevalence of such conditions was low in girls with a questionnaire available, and including or excluding them had little impact on the resulting estimates (data not shown).

In conclusion, we found that US and Tanner B staging are in close agreement when determining the degree of pubertal breast maturation. US allows for a direct examination of the mammary gland and detects pubertal breast development slightly ahead of clinical Tanner B staging. Moreover, this method allows to discriminate 2 distinct prepubertal stages, the clinical relevance of which will be the subject of further research. Pubertal breast and PH development in Norwegian girls occur at comparable ages as in their northern European peers. In girls of Norwegian origin, age at menarche occurred at a significantly younger age than 10 years before. This trend toward earlier menarche merits further investigation.

## Abbreviations

BGS 1: Bergen Growth Study 1
BGS 2: Bergen Growth Study 2
US B: ultrasound breast stage
Tanner B: Tanner breast stage
Tanner PH: Tanner pubic hair stage

## Notes

***Financial Support:*** This study was supported by a grant (number 912221) from the Western Norway Regional Health Authority.

## Additional Information

***Disclosure Summary:*** The authors have nothing to disclose.

***Data Availability:*** Restrictions apply to the availability of data generated or analyzed during this study to preserve patient confidentiality or because they were used under license. The corresponding author will on request detail the restrictions and any conditions under which access to some data may be provided.
